# Impact of interaction between the G870A and EFEMP1 gene polymorphism on glioma risk in Chinese Han population

**DOI:** 10.18632/oncotarget.16581

**Published:** 2017-03-27

**Authors:** Libin Yang, Bo Qu, Xun Xia, Yongqin Kuang, Jian Li, Kexia Fan, Heng Guo, Hui Zheng, Yuan Ma

**Affiliations:** ^1^ Department of Neurosurgery, Chengdu Military General Hospital, Chengdu, Sichuan 610083, China; ^2^ Department of Anatomy, Chengdu Medical College, Chengdu, Sichuan 610500, China

**Keywords:** CCND1, EFEMP1, glioma, SNP, interaction

## Abstract

**Aims:**

To investigate the impact of CCND1 and EFEMP1 gene polymorphism, and additional their gene-gene interactions and haplotype within EFEMP1 gene on glioma risk based on Chinese population.

**Methods:**

Logistic regression was performed to investigate association between single-nucleotide polymorphisms (SNP) and glioma risk and generalized multifactor dimensionality reduction (GMDR) was used to analyze the gene-gene interaction.

**Results:**

Glioma risks were higher in carriers of homozygous mutant of rs603965 within CCND1 gene, rs1346787 and rs3791679 in EFEMP1 gene than those with wild-type homozygotes, OR (95%CI) were 1.67 (1.23-2.02), 1.59 (1.25-2.01) and 1.42 (1.15-1.82), respectively. GMDR analysis indicated a significant two-locus model (p=0.0010) involving rs603965 within CCND1 gene and rs1346787 within EFEMP1 gene. Overall, the cross-validation consistency of the two- locus models was 10\ 10, and the testing accuracy is 60.17%. Participants with rs603965 - GA or AA and rs1346787- AG or GG genotype have the highest glioma risk, compared to participants with rs603965 - GG and rs1346787- AA genotype, OR (95%CI) was 3.65 (1.81-5.22). We conducted haplotype analysis for rs1346787 and rs3791679, because D′ value between rs1346787 and rs3791679 was more than 0.8. The most common haplotype was rs1346787 – A and rs3791679- G haplotype, the frequency of which was 0.4905 and 0.4428 in case and control group.

**Conclusions:**

Polymorphism in rs603965 within CCND1 gene and rs1346787 within EFEMP1 gene and its gene- gene interaction were associated with increased glioma risk.

## INTRODUCTION

Glioma is the most common tumor of the central nervous system in adults, accounting for more than 70% of all types of brain tumor [1]. The 5-year survival probability of patients with glioma varies by subtype, but is as low as 4.7% [2]. In 2000, the annual incidence of cerebral tumors was about 3.9 per 100,000 in men and 2.8 per 100,000 in women among Chinese population, also 2–3 per 100,000 in European and North America [3, 4]. The molecular mechanism, etiology, and epidemiology of glioma are not well understood. Previously, some risk factors have been reported in several studies, including workplace, diet, and other personal or residential exposures and genetic factors [5, 6]. Recent years, the genetic factors including single nucleotide polymorphisms (SNPs) may also exert effects on the development of glioma [5], including Cyclin D1 (CCND1) and EFEMP1 gene [7–9]. Understanding the genetic etiology of gliomas may help to reveal the mechanism of gliomas and provide new insight for the diagnosis and treatment.

CCND1 is a cell cycle regulatory gene located at chromosome 11q13, which is responsible for G1–S transition regulation during cell cycle [9]. Previously, some studies focused on the susceptibility of the Cyclin D1 (CCND1) G870A gene polymorphism to several type of cancer, including bladder cancer, breast cancer, colorectal cancer, leukemia and lung cancer [10–13]. However, the potential correlation between the CCND1 G870A polymorphism and glioma risk is not clear yet, till now, limited population based studies have been conducted to investigate the association between CCND1 G870A polymorphism and glioma risk. EFEMP1, as known as fibulin-3, is located in chromosome 2 and encodes a member of the fibulin family of extracellular matrix glycoproteins. Previous studies have revealed that EFEMP1 played a role in the nature of many malignant tumors and interacted with its partners [14, 15]. Several studies have indicated that he over-expression of EFEMP1 could inhibit the tumorigenicity of fibrosarcoma cells, while down- expression of EFEMP1 or EFEMP1 promoter methylation occurred in many cancers [16], and played important promoting or inhibiting growth roles in cancers [17], however, till now, just one study [9] has examined the susceptibility of EFEMP1 gene with diseases.

The multiple genotypes, such as glioma, was a result of many gene polymorphism, gene- gene interactions, considering CCND1 G870A polymorphism and EFEMP1both are risk factor of glioma in previous literatures, but no study focused on this the interaction between CCND1 G870A polymorphism and EFEMP1 polymorphisms. In addition, the implication of LD in association studies is that knowledge of variation at a certain position also gives knowledge of variation at linked loci. So in this study, we aimed to the impact of CCND1 and EFEMP1 gene polymorphism, and additional their gene-gene interactions and haplotype within EFEMP1 gene on glioma risk based on Chinese population.

## RESULTS

A total of 1056 participants (583 men, 523 women) with a mean age of 51.7 ± 9.8 years old, were selected, including 350 glioma patients and 706 normal controls. Table 1 shows the participants characteristics in cases and controls. The mean of age and distribution of males, smoking and alcohol drinking were not different in glioma cases and controls. The rate of family history of cancer was higher in cases than that in controls. In glioma cases, there are 123 cases with glioblastoma and 227 cases with low-grade gliomas.

**Table 1:** General characteristics of study participants in glioma cases and controls

Variables	Cases (n=350)	Controls (n=706)	*p-values*
Age (years)	51.3±10.8	52.1±10.4	0.246
Males N (%)	187(53.4)	402(56.9)	0.279
Alcohol drinking N (%)	121(34.6)	252(35.7)	0.719
Smoke N (%)	100 (28.6)	206(29.2)	0.838
Family history of cancer N (%)	118(33.7)	115 (16.3)	<0.001
Histological types N (%)			
Glioblastoma N (%)	123 (35.1)		
Low-grade gliomas N (%)	227(64.9)		

Abbreviations: N, number.

Table 2 shows the frequencies of alleles and genotypes within four SNPs in cases and controls. All genotypes were distributed according to Hardy–Weinberg equilibrium in controls (P>0.05). Logistic analysis showed the significant association of rs603965 within CCND1 gene, rs1346787 and rs3791679 in EFEMP1 gene with increased glioma risk, after adjustment for gender, age, smoking and alcohol drinking. Glioma risks were higher in carriers of homozygous mutant of rs603965 within CCND1 gene, rs1346787 and rs3791679 in EFEMP1 gene than those with wild-type homozygotes, OR (95%CI) were 1.67 (1.23-2.02), 1.59 (1.25-2.01) and 1.42 (1.15-1.82), respectively.

**Table 2:** Logistic analysis on the association between 4 SNPs and glioma risk

SNPs	Genotypes and alleles	Frequencies N (%)		OR (95%CI)*	H-W test
		Cases (n=350)	Controls(n=706)		
CCND1					
rs603965 G870A					
	Additive				
	GG	162(46.3)	436(61.8)	1.00	0.674
	GA	142(40.6)	240(34.0)	1.48(1.16-1.82)	
	AA	46(13.1)	30(4.2)	2.16(1.32-2.94)	
	Dominant				
	GG	162(46.3)	436(61.8)	1.00	
	GA+AA	188(53.7)	270(38.2)	1.67(1.23-2.02)	
	Allele, A (%)	234(33.4)	300(21.2)		
EFEMP1					
rs1346787					
	Additive				
	AA	157(44.9)	414(58.6)	1.00	0.193
	AG	150(42.9)	245 (34.7)	1.52(1.21 -1.88)	
	GG	43(12.2)	47(6.7)	1.95(1.36-2.66)	
	Dominant				
	AA	157(44.9)	414(58.6)	1.00	
	AG+ GG	193(55.1)	292(41.4)	1.59(1.25-2.01)	
	Allele, G (%)	236(33.7)	339(24.0)		
rs3791679					
	Additive				
	GG	170(48.6)	444(62.9)	1.00	0.228
	GA	139(39.7)	225(31.9)	1.31(1.08-1.67)	
	AA	41(11.7)	37(5.2)	1.88(1.34-2.42)	
	Dominant				
	GG	170(48.6)	444(62.9)		
	GA+AA	180(51.4)	262(37.1)	1.42(1.15-1.82)	
	Allele, A (%)	221(31.6)	299(21.2)		
rs17047290					
	Additive				
	AA	197(56.3)	427(60.5)	1.00	0.851
	AG	126(36.0)	243(34.4)	1.12(0.94-1.47)	
	GG	27(7.7)	36(5.1)	1.38(0.90-1.95)	
	Dominant				
	AA	197(56.3)	427(60.5)	1.00	
	AG+ GG	153(43.7)	279(39.5)	1.18(0.93-1.62)	
	G	179(25.6)	315(22.3)		

^a^ Adjustment for gender, age, smoking and alcohol drinking.

We employed the GMDR to screen the significant gene-gene interaction combination. Table 3 summarizes the results obtained from GMDR analysis from two- to four-locus models. We found a significant two-locus model (p=0.0010) involving rs603965 within CCND1 gene and rs1346787 within EFEMP1 gene. Overall, the cross-validation consistency of the two- locus models was 10\ 10, and the testing accuracy is 60.17%. We also conducted interaction analysis between rs603965 within CCND1 gene and rs1346787 within EFEMP1 gene by using logistic regression. We found that participants with rs603965 - GA or AA and rs1346787- AG or GG genotype have the highest glioma risk, compared to participants with rs603965 - GG and rs1346787- AA genotype, OR (95%CI) was 3.65 (1.81-5.22), after adjustment for covariates including gender, age, smoking and alcohol drinking. (Table 4)

**Table 3:** Best gene–gene interaction models, as identified by GMDR

Locus no.	Best combination	Cross-validation consistency	Testing accuracy	*p-values* ^a^
2	rs603965 rs1346787	10/10	0.6017	0.0010
3	rs603965 rs1346787 rs3791679	9/10	0.5399	0.0547
4	rs603965 rs1346787 rs3791679 rs17047290	7/10	0.4958	0.1719

^a^ Adjustment for gender, age, smoking and alcohol drinking.

**Table 4:** Interaction between rs603965 and rs1346787 on glioma risk

rs603965	rs1346787	OR (95% CI)^a^	*P*-values
GG	AA	1.00	-
GG	AG or GG	1.52 (1.19-2.04)	0.001
GA or AA	AA	1.47 (1.16-1.91)	0.002
GA or AA	AG or GG	3.65 (1.81-5.22)	<0.001

^a^ Adjustment for gender, age, smoking and alcohol drinking.

Pairwise LD analysis between SNPs within EFEMP1was measured, and D′ value between rs1346787 and rs3791679 was 0.814, D′ value between rs1346787 and rs17047290 was 0.526, D′ value between rs3791679 and rs17047290 was 0.518. We conducted haplotype analysis for rs1346787 and rs3791679, because D′ value between rs1346787 and rs3791679 was more than 0.8. The most common haplotype was rs1346787 - A- rs3791679- G haplotype, the frequency of which was 0.4905 and 0.4428 in case and control group. However, we did not find any haplotype combination statistically significantly associated with glioma risk (Table 5), after adjustment for gender, age, smoking and alcohol drinking.

**Table 5:** Haplotype analysis on association between EFEMP1 and glioma risk

Haplotypes	rs1346787	rs3791679	Frequencies		OR(95%CI)	*p*-values*
			Control group	Case group		
H1	**A**	G	0.4905	0.4428	1.00	--
H2	**G**	G	0.2688	0.2809	1.22(0.95 - 1.67)	0.090
H3	**A**	A	0.1858	0.2095	1.33 (0.89 - 1.86)	0.528
H4	**G**	A	0.0549	0.0668	1.29 (0.98 – 1.65)	0.083

^a^ Adjustment for gender, age, smoking and alcohol drinking.

## DISCUSSION

In this study based on Chinese Han population, we found a significant association between rs603965 within CCND1 gene, rs1346787 and rs3791679 within EFEMP1 gene and increased glioma risk, after covariates adjustment. Excessive CCND1 expression with or without biological activity is common in human cancers. The G870A mutation is one of the most common SNPs of this gene CCND1gene. Although the CCND1 G870A polymorphism has been investigated in a number of cancers [10–13], there are few studies on the association of the CCND1 G870A polymorphism with glioma risk and the potential correlation between the CCND1 G870A polymorphism and glioma risk is not clear yet, till now, limited population based studies have been conducted to investigate the association between CCND1 G870A polymorphism and glioma risk. Qin et al [18] conducted a meta- analysis and indicated that CCND1 G870A polymorphism may increase brain tumor risk, especially for gliomas, and they also suggested that more primary large scale and well-designed studies are still required to evaluate the interaction of CCND1 G870A polymorphism with brain tumor risk. Results of a study by Zeybek et al [19] indicate a possible relation between GBM formation and CCDN1 genotype. Chen et al [8] conducted a case- control study for Chinese population and suggested that the CCND1 G870A polymorphism modulated oncogenic cyclin D1b expression in glioma tissues and may be associated with an increased risk of gliomas in Chinese population. Another meta- analysis [7] study indicated that meta-analysis suggests that the CCND1 G870A polymorphism may play a important role in the development of glioma, but the currently available data pooled in their meta-analysis are limited so the precise association of CCND1 G870A polymorphism with glioma risk needs further elucidation, and future studies with a larger sample size are needed to investigate the possible effects of gene–gene interaction in the glioma risk detection. Recently, Liu et al [20] conducted a simple study in Chinese population and suggested that the CCND1 G870A polymorphism may contribute to the susceptibility to glioma in Chinese population. However, the sample of this study was also relatively small, so study with larger sample were needed to verify this association. And in current study, we obtained the similar results with previous studies and the sample of this study was relatively larger than fore- mentioned studies.

EFEMP1, was another gene which has been reported association with glioma risk recently. Several studies have indicated that he over-expression of EFEMP1 could inhibit the tumorigenicity of fibrosarcoma cells, while down- expression of EFEMP1 or EFEMP1 promoter methylation occurred in many cancers [16], and played important promoting or inhibiting growth roles in cancers [17], however, till now, just one population- based study [9] has examined the susceptibility of EFEMP1 gene with diseases. Zhang et al [9] conducted a hospital-based case–control study in a Chinese Han population and suggested that common genetic variants in EFEMP1 gene were associated with glioma and contributed to glioma susceptibility, which might help to reveal the mechanism of gliomas and provide new insight for the diagnosis and treatment. In this study, they found that rs1346787, rs3791679, rs1346786 and rs3791675 were all associated with increased glioma risk. The results about the function of EFEMP1 in glioma were conflicting. Hu et al [21] reveals that EEFEMP1 suppresses glioma growth *in vivo*, both by modulating the tumor extracellular microenvironment and by altering critical intracellular oncogenic signaling pathways. Previously, no functional relevance of the rs1346786 and glioma was reported. It is possible that this SNP might increase the affinity of transcription activators or decrease that of transcription suppressors to the intron enhancer, thus up- regulating the expression levels of EFEMP1. Another SNP (rs1346787), which located in the 3′near gene, might be associated with the regulation of transcription termination and then effect the transcription and translation.

The multiple genotypes, such as glioma, was a result of many gene polymorphism, gene- gene interactions, considering CCND1 G870A polymorphism and EFEMP1both are risk factor of glioma in previous literatures, but no study focused on this the interaction between CCND1 G870A polymorphism and EFEMP1 polymorphisms. In addition, the implication of LD in association studies is that knowledge of variation at a certain position also gives knowledge of variation at linked loci. So in this study, we investigated the impact of interaction between CCND1 and EFEMP1 gene polymorphism on glioma risk. We found a significant two-locus model (p=0.0010) involving rs603965 within CCND1 gene and rs1346787 within EFEMP1 gene, participants with rs603965 - GA or AA and rs1346787- AG or GG genotype have the highest glioma risk, compared to participants with rs603965 - GG and rs1346787- AA genotype. The results of this study suggest that CCND1 G870A genetic variants may modify the influence of EFEMP1- rs1346787 gene on glioma risk. The mechanism of this interaction was not well known, and maybe this combined or crossover effect on some glioma- related risk factors could lead to the interaction between PPAR G and CYP1A1 gene on CAD risk. A connection between CCND1 G870A and EFEMP1 agonist treatment may become apparent in the future agonist studies. As we all know that, Loci that are located nearby on the same chromosome may be in LD. This means that alleles at these loci are not inherited in an independent manner but certain allele combinations occur more often than expected by random segregation. The implication of LD in association studies is that knowledge of variation at a certain position also gives knowledge of variation at linked loci. In this study, pairwise LD analysis between SNPs within EFEMP1was measured, and just D′ value between rs1346787 and rs3791679 was more than 0.8, then we conducted haplotype analysis for rs1346787 and rs3791679. We did not found any haplotype combination statistically significantly associated with glioma risk. Zhang et al [9] also conducted haplotype analysis for SNPs within EFEMP1 gene, and they also found no significant haplotype combination in the study.

Several limitations of this study should be considered. Firstly, the sample of this study was relatively small, more participants in different races should been included in the study; Secondly, gene- environment interaction with some environmental risk factors should be investigated in the future studies, such as hypertension, diabetes and so on. Thirdly, the function of these SNPs within CCND1 and EFEMP1 gene should be investigated in cell line or animal model.

In conclusion, we found a significant we found a significant association between rs603965 within CCND1 gene, rs1346787 and rs3791679 within EFEMP1 gene and increased glioma risk. We also found a significant two-locus model (p=0.0010) involving rs603965 within CCND1 gene and rs1346787 within EFEMP1 gene, participants with rs603965 - GA or AA and rs1346787- AG or GG genotype have the highest glioma risk, compared to participants with rs603965 - GG and rs1346787- AA genotype. But we did not found any haplotype combination statistically significantly associated with glioma risk.

## MATERIALS AND METHODS

### Subjects

This was a hospital based case-control study. Participants were consecutively recruited between 26 June 2010 and 15 March 2015, a total of 353 patients histopathologically diagnosed with gliomas were recruited from Chengdu Military General Hospital, excluded 3 patients who refused for taking part in this investigation, 350 gliomas patients were included in this study. Control subjects were randomly selected from trauma outpatients and annual check-up visitors at the same hospital during the similar time period. The controls with a self-reported history of cancer or central nervous system-related diseases and previously receiving radiotherapy/chemotherapy for certain diseases were excluded, a total of 706 participants were included in control group in this study. At recruitment, written informed consent was obtained from all participants. All participants were interviewed by trained nurses using questionnaire to collect detailed demographic information, lifestyle and family history of cancer data. We defined currently alcohol consumption as more than 1 drink of any type per month or not currently drinking as less than 1 drink of any type per month; Current smokers were defined as those who have smoked for at least 100 cigarettes and still smoked at the time of the interview, individuals with no history of cigarette smoking were considered as never smokers. Blood samples were collected in the morning after at least 8 hours of fasting. All plasma and serum samples were frozen at –80°C until laboratory testing.

### Genomic DNA extraction and genotyping

The rs603965 (G870A) mutation within CCND1 gene was selected because it is one of the most common SNPs of this gene and have been more studied. A total of three SNPs in EFEMP1 gene are selected for genotyping in the study: rs1346787, rs3791679 and rs17047290, which were located in the same block. Peripheral venous blood was drawn from each participant. Genomic DNA from participants was extracted from EDTA-treated whole blood, using the DNA Blood Mini Kit (Qiagen, Hilden, Germany) according to the manufacturer's instructions. A870G was detected by polymerase chain reaction-restriction fragment length polymorphisms (PCR-RFLP), for genotyping, a restriction enzyme was used to identify and cut specific sequences, after which PCR was performed with the primers, which shown in Table 6. PCR cycling conditions were as following: one cycle at 94°C for 5 min; 35 cycles of 94°C for 30 s, 55°C for 30 s, and 72°C for 30 s, and a final extension at 72°C for 10 min. rs1346787, rs3791679 and rs17047290 within EFEMP1 gene were detected by Taqman fluorescence probe. Probe sequences of these three SNPs were shown in Table 6. ABI Prism7000 software and allelic discrimination procedure was used for genotyping. A 25μl reaction mixture including 1.25ul SNP Genotyping Assays (20×), 12.5μl Genotyping Master Mix (2×), 20 ng DNA, and the conditions were as follows: initial denaturation for 9 min and 94°C, denaturation for 18 s and 93°C, annealing and extension for 80 s and 62°C, 50 cycles.

**Table 6:** Description and primer/probe sequence used for genotyping for 4 SNPs

ID	Gene name	Chromosome	Functional Consequence	Nucleotide substitution	Primer sequences/Probe sequence
rs603965 G870A	CCND1	11:69648142	Synonymous codon	G>A	Forward:5’-GTGAAGTTCATTTCCAATCCGC-3’ Reverse: 5’-GGG ACA TCACCCTCACTTAC-3’.
rs1346787	EFEMP1	2:55865477	downstream variant 500B, intron variant	A>G	5’-TCATTCCTCTTACCCTGTCTACATG[A/G] TTGCTTACAGTCAAGATAAATTACA-3’
rs3791679	EFEMP1	2:55869757	Intron variant	G>A	5’-TAACTGTTTCATGCTAATGATGTTA[G/A] TCATCCAGTTACAATTTTCTCAAAA-3’
rs17047290	EFEMP1	2:55878799	Intron variant	A>G	5’-TGGCAATTACTAGCTTGGTCTTAAC[C/T] TCTCGAGCTTGCATTTCCTCTTCTG-3’

### Statistical analysis

The mean and SD were calculated for normally distributed continuous variables, and percentages were calculated for categorical variable. The categorical data were analyzed using χ^2^ test. Further, continuous variables were analyzed using Student's t test. Hardy-Weinberg equilibrium (HWE) was performed by using SNPStats (available online at http://bioinfo.iconcologia.net/SNPstats). Generalized multifactor dimensionality reduction (GMDR) model was used to analyze the gene- gene interaction, cross-validation consistency, the testing balanced accuracy, and the sign test, to assess each selected interaction were calculated. Logistic regression was performed to investigate association between SNPs and gliomas using gender, age, smoking and alcohol drinking as covariates in the model.
